# Inspissated Bile Syndrome and Crigler–Najjar Syndrome Type II: When Two Rare Conditions Converge

**DOI:** 10.1155/crpe/4964181

**Published:** 2025-10-06

**Authors:** Razieh Sangsari, Maryam Saeedi, Kayvan Mirnia, Fatemeh Tafreshi

**Affiliations:** ^1^Department of Pediatrics, Division of Neonatology, School of Medicine, Children's Medical Center, Tehran University of Medical Sciences, Tehran, Iran; ^2^Department of Pediatrics, School of Medicine, Children's Medical Center, Tehran University of Medical Sciences, Tehran, Iran

**Keywords:** Crigler–Najjar syndrome, inspissated bile syndrome, newborn, pregnant women

## Abstract

**Background:**

Inspissated bile syndrome (IBS) is a rare neonatal condition characterized by thickened bile leading to cholestasis, while Crigler–Najjar syndrome type II (CNS-II) is an autosomal recessive disorder resulting in unconjugated hyperbilirubinemia. Both conditions pose significant risks, particularly when they converge during pregnancy.

**Case Presentation:**

We report a unique case involving a 20 day-old female neonate born to a mother with CNS-II who discontinued phenobarbital treatment during pregnancy. The mother presented with elevated indirect bilirubin levels (total 32, and direct 0.4 mg/dL) and normal liver function test, while the neonate exhibited prolonged jaundice and cholestasis with direct hyperbilirubinemia (total 8, and direct 5.4 mg/dL) upon admission. Treatment included ursodeoxycholic acid for the neonate, leading to normalization of bilirubin levels. The neonate's growth and neurological development remained normal at 6 months.

**Discussion:**

This case emphasizes the significant risks associated with untreated CNS-II during pregnancy, which likely contributed to the neonate's IBS. Existing literature highlights the importance of maternal management in preventing severe neonatal complications associated with hyperbilirubinemia.

**Conclusion:**

This case highlights the importance of timely intervention in managing maternal metabolic disorders during pregnancy to improve neonatal health. Further research is essential to develop optimal treatment strategies for such cases.

## 1. Introduction

Inspissated bile syndrome (IBS) is a rare and significant condition in neonates, characterized by the thickening and stasis of bile within the biliary system. This leads to cholestasis and potential liver damage. IBS can be particularly challenging to manage in neonates, especially those born to mothers with underlying metabolic disorders, such as Crigler–Najjar syndrome type II (CNS-II) [1]. CNS-II is an autosomal recessive disorder caused by mutations in the uridine diphospho-glucuronosyltransferase 1A1 (UGT1A1) gene, resulting in a partial deficiency of the enzyme uridine diphosphate-glucuronosyltransferase (UDPGT). This enzyme deficiency results in unconjugated hyperbilirubinemia, which can pose significant risks for the neonates, such as kernicterus, particularly if the bilirubin levels are elevated during the perinatal period [2].

CNS is classified into two types based on the severity of the enzyme deficiency. Type I is characterized by a complete absence of UDPGT activity, leading to severe, persistent unconjugated hyperbilirubinemia that can result in kernicterus and death if untreated. Type II, also known as Arias syndrome, involves a partial deficiency of the enzyme, resulting in less severe hyperbilirubinemia that can often be managed with phenobarbital, which induces the activity of the remaining enzyme [3]. The management of CNS-II during pregnancy is complex and requires careful monitoring and treatment to prevent adverse outcomes for both the mother and the fetus. The studies on CNS-II in pregnancy emphasize the importance of phenobarbital therapy in reducing maternal bilirubin levels and preventing fetal complications [4–7].

The case presented in this report is unique because the mother with CNS-II discontinued her medications, including phenobarbital, during her pregnancy. This lack of treatment poses significant risks, as untreated CNS-II can lead to elevated maternal bilirubin levels, which may cross the placenta and result in fetal hyperbilirubinemia and kernicterus [8]. Consequently, the neonate in this case developed IBS, a condition that can arise from severe hyperbilirubinemia and cholestasis.

## 2. Case Presentation

A 20-day-old female infant, born at 39 weeks of gestational age, birth weight of 3200 g, and admission weight 3600 g to a mother with a history of CNS-II, presented to our hospital with prolonged jaundice and cholestasis. Upon admission, the neonate was found to have a history of cholestasis since birth, but no other significant abnormalities were noted.

Laboratory tests revealed mild elevated liver enzymes, with a serum gamma-glutamyl transferase (GGT) level of 385 IU/L, a total bilirubin level of 8.0 mg/dL, and a direct bilirubin level of 5.4 mg/dL. Blood and urine cultures were negative, and C-reactive protein, blood indexes, normal feeding, and normal primitive reflexes ruling out sepsis. Metabolic screening using mass spectrometry was normal. The blood type of the mother and the baby was B+, and hemolysis was ruled out due to the lack of hemoglobin drop and the absence of reticulocytosis in the newborn. An abdominal ultrasound showed moderate gallbladder sludge and a borderline increase in the thickness of the common bile duct. Although the mother used phenobarbital treatment before pregnancy and her jaundice was under control, she stopped taking the medicine during pregnancy due to fears about its safety and then the bilirubin level rose to 32 mg/dL.

Given the neonate's history of cholestasis, elevated direct bilirubin levels, rule out of congenital, metabolic, and infectious causes, and abnormal ultrasound findings, a diagnosis of IBS was considered. This condition is characterized by thickened and hardened bile within the biliary tree, leading to obstruction and jaundice. The mother was treated with phenobarbital, while the neonate was treated with ursodeoxycholic acid, a medication used to dissolve bile and improve bile flow. Additionally, the neonate received fat-soluble vitamins at twice the essential required dose for their age, as well as water-soluble vitamins at the basic required dose for their age to address any potential malabsorption issues. After several weeks of treatment, the neonate's jaundice improved, and liver enzymes, serum GGT, and bilirubin levels normalized. To assess for bilirubin encephalopathy, an auditory brainstem response (ABR) was conducted in the neonate, which returned normal results. The neonate was followed until six months of age, the growth was normal range in the 50th percentile, and developmental examinations were normal using the Ages and Stages Questionnaire, Third Edition (ASQ-3).

## 3. Discussion

This case report presents a neonate born to a mother with CNS-II, who had been treated with phenobarbital prior to pregnancy but did not receive during her pregnancy. The absence of maternal treatment for CNS-II during pregnancy is particularly noteworthy, as it significantly impacts the neonatal outcome [9]. CNS-II is characterized by a partial deficiency in the enzyme UDPGT, leading to moderate-to-severe unconjugated hyperbilirubinemia. Typically, phenobarbital is used to induce UDPGT activity and reduce bilirubin levels in affected individuals. However, in this case, the mother did not receive phenobarbital during her pregnancy, which likely contributed to the development of unconjugated hyperbilirubinemia in the fetus, because maternal unconjugated bilirubin, due to its lipid-soluble nature, readily crosses the placenta and result in fetal hyperbilirubinemia [8]. On the other hand, the load of conjugation of unconjugated bilirubin by neonate produces obstruction of bile flow due to thick and inspissated bile within the biliary system who presented with cholestasis [10]. The only significant finding in our infant was cholestasis, which fully responded to ursodeoxycholic acid treatment during the follow-up after discharge. ABR was normal, and ASQ3 and the neurodevelopmental examination at 6 months of age were also normal. Maternal unconjugated hyperbilirubinemia raises concerns regarding fetal neurodevelopment, especially during late gestation, when the fetal blood–brain barrier remains immature and susceptible. Elevated maternal bilirubin may expose the fetus to concentrations that could interfere with neuronal maturation, synaptogenesis, or even increase the risk of bilirubin-induced neurological dysfunction under certain conditions. Although reported outcomes in CNS-II pregnancies have generally been favorable, the absence of large-scale longitudinal studies leaves significant uncertainty regarding subclinical or long-term developmental effects. The potential for fetal bilirubin accumulation—and its theoretical neurotoxicity—should therefore not be dismissed [5]. Treatment during pregnancy for women with CNS-II is centered on managing bilirubin levels to protect both the mother and the baby—especially the newborn, who is at high risk for acute bilirubin toxicity immediately after birth. Phenobarbital plays a critical role in this process. It boosts the mother's ability to conjugate bilirubin, which helps lower circulating levels and minimizes fetal exposure. Despite common fears about its safety, evidence shows that phenobarbital—at the low doses typically used for CNS-II—is generally safe during pregnancy that makes consistent use of this medication essential, even if some patients feel uncertain about side effects or long-term use. The transition to postpartum care is equally vital. Newborns may experience a sharp surge in unconjugated bilirubin due to their underdeveloped enzyme systems. This calls for rapid intervention—such as phototherapy or more intensive measures—to avoid permanent neurological damage. Proactive neonatal care teams must be ready to act swiftly within the first hours of life [11].

A case report described a 37-year-old mother with CNS-II who was treated with phenobarbital during her pregnancy. This treatment resulted in controlled bilirubin levels, and she gave birth to a healthy neonate who presented with mild hyperbilirubinemia that resolved without intervention [5]. Another reports detailed the successful use of phototherapy and phenobarbital in a pregnant woman with CNS-II, which significantly reduced her bilirubin levels and led to the birth of a healthy child [7, 12, 13].

By examining this case, we aim to enhance the understanding of the interplay between maternal metabolic disorders and neonatal liver conditions. Furthermore, we seek to underscore the importance of appropriate maternal treatment in preventing adverse neonatal outcomes. The findings from this case may offer valuable insights for clinicians managing similar situations, highlighting the necessity for multidisciplinary care to optimize both maternal and neonatal health outcomes.

## 4. Conclusion

In conclusion, this case underscores the importance of early and effective management of hyperbilirubinemia in pregnant women with CNS-II to ensure better neonatal health outcome. Further research is needed to explore optimal treatment strategies for pregnant women with CNS-II to prevent neonatal complications.

## Data Availability

The authors confirm that all data are included in this study are available from the corresponding author upon on reasonable request.
